# A10 TIME-TRAJECTORY ANALYSIS OF PROTEOMICS REVEALS POTENTIAL PRE-CLINICAL STAGES OF CROHN’S DISEASE

**DOI:** 10.1093/jcag/gwaf042.010

**Published:** 2026-02-13

**Authors:** R Chen, W Turpin, A Griffiths, H Steinhart, H Q Huynh, K Jacobson, S Murthy, K Croitoru, S Lee

**Affiliations:** IBD Center and Zane Cohen Centre for Digestive Diseases, Lunenfeld-Tanenbaum Research Institute, Toronto, ON, Canada; IBD Center and Zane Cohen Centre for Digestive Diseases, Lunenfeld-Tanenbaum Research Institute, Toronto, ON, Canada; The Hospital for Sick Children, Toronto, ON, Canada; Mount Sinai Hospital, Toronto, ON, Canada; Stollery Children’s Hospital, University of Alberta, Edmonton, AB, Canada; BC Children’s Hospital, Vancouver, BC, Canada; University of Ottawa, Ottawa, ON, Canada; IBD Center and Zane Cohen Centre for Digestive Diseases, Lunenfeld-Tanenbaum Research Institute, Toronto, ON, Canada; IBD Center and Zane Cohen Centre for Digestive Diseases, Lunenfeld-Tanenbaum Research Institute, Toronto, ON, Canada

## Abstract

**Background:**

Previous studies have identified multiple biomarkers that precede the diagnosis of Crohn’s disease (CD). However, the time trajectory of biological events towards diagnosis remain poorly understood.

**Aims:**

To map the molecular stages of pre-clinical CD and develop a proteomics-based risk score for predicting the time trajectory toward CD onset.

**Methods:**

We conducted a nested-case control study in two prospective cohorts: the GEM project (healthy first-degree relatives of CD patients; n = 521), and the UK biobank (n = 720); each case of incident CD (pre-CD) was matched to healthy controls by demographic characteristics and follow-up time. Serum proteomics were profiled using a Olink®-HT panel (5,416 proteins) in GEM. Conditional logistic regression was conducted to assess the association between the level of proteins and CD risk. Estimated trajectory analysis was performed to evaluate the ‘dynamic change’ (based on the slope of protein level change across time in the pre-CD vs controls) and the ‘variation timepoint’ (when the protein level begins to diverge between pre-CD vs controls) of proteins. Functional pathway analysis was performed using GO analysis. A Proteomics Risk Score, using machine learning-based algorithm, was trained on 70% subset of the GEM cohort and validated in the remaining 30% (testing cohort).

**Results:**

In the GEM cohort (105 pre-CD cases, 416 matched controls), we identified 73 proteins associated with CD onset (difference of protein levels in pre-CD vs controls) and 108 with dynamic changes (slope difference in pre-CD vs controls) preceding diagnosis; 87.1% and 68.4% of these proteins, respectively, had consistent direction in the UK biobank (120 pre-CD cases, 600 matched controls). These validated proteins were reclassified as: Near onset (variation timepoint<2 years prior diagnosis, n = 21); Early stage (variation timepoint 2-4 years prior diagnosis, n = 28); Parallel change (proteins significant in association analysis but with no dynamic changes, n = 63). Functional pathway analysis revealed pathways enriched in host-microbe interaction, extracellular matrix organization and barrier integrity, and innate immunity in “Near” onset, Early stage, and Parallel change proteins, respectively. Furthermore, a Proteomics Risk Score, consisting of parallel and dynamic change proteins, demonstrated an excellent performance to predict CD onset (AUC: 0.806) and strongly correlated with time to diagnosis (r = 0.552, P = 0.0013) in the testing cohort.

**Conclusions:**

Proteomic signatures with distinct time-trajectories may define molecular stages in the pre-disease phase of CD. A proteomics risk score derived from these proteins enables stratification of individuals along this pre-disease timeline, offering a framework for risk prediction and potential early intervention in CD.

**Funding Agencies:**

CCC, CIHRHemsley Charitable Trust

